# Department managers’ perceptions of a priority-setting model in a local healthcare organisation: a mixed-methods study

**DOI:** 10.1186/s12913-026-14451-z

**Published:** 2026-03-31

**Authors:** Inga-Britt Gustafsson, Lars Wallin, Ulrika Winblad, Mio Fredriksson

**Affiliations:** 1 Department of Public Health and Caring Sciences, Health Services Research, Uppsala University, Box 564, Uppsala, 751 22 Sweden; 2 Centre for Clinical Research, Nissers väg 3, Dalarna, Falun, 791 82 Sweden; 3 Dalarna University, Falun, 791 88 Sweden

**Keywords:** Decommissioning, Legitimacy, Local healthcare organisation, Mixed method, Priority-setting framework

## Abstract

**Background:**

Priority-setting models have the potential to enhance transparency and support decision-making under conditions of resource scarcity; however, their practical application remains limited, particularly in large-scale decommissioning programmes. This article examines department managers’ perceptions of using a National Model for Transparent Prioritization (NMTP) at their clinics during the implementation of a decommissioning programme. In addition, the study explores whether the model contributes to fair priority setting and to procedural and substantive legitimacy.

**Methods:**

A convergent mixed-methods design was employed, integrating quantitative and qualitative data from a survey (*n* = 33) and semi-structured interviews (*n* = 22). The analysis addressed six questions grouped into two overarching themes: (1) the model’s use and contribution (adoption, exclusion, and inclusion of care) and (2) the model’s legitimacy (acceptance, justification, and fairness of priorities).

**Results:**

Department managers expressed varying perceptions of the NMTP. Approximately half of them considered the time and resources required to be justified and perceived that the model ensured patients with the greatest needs were given the highest priority. About one third agreed that the NMTP was accepted as a tool for prioritization and resource allocation, and a similar proportion reported that the NMTP had been used to exclude care that was previously provided.

**Conclusions:**

Department managers perceived that the NMTP facilitated the application of the Swedish ethical guidelines; however, they also identified several challenges associated with its use in the context of a large-scale decommissioning programme, including time constraints, a substantial initial threshold, demanding evidence requirements, and insufficient alignment with national objectives. Furthermore, resistance encountered within clinical units may have been attributable to limited stakeholder engagement.

**Supplementary Information:**

The online version contains supplementary material available at 10.1186/s12913-026-14451-z.

## Introduction

Priority-setting procedures can be understood as a specific approach to managing resource scarcity and budgetary constraints in healthcare organizations [1]. Priority-setting procedures operate at multiple levels of healthcare systems, ranging from national health technology assessments and evidence-based clinical guidelines to organizational decision frameworks for resource allocation and frontline clinical triage processes [1–3]. While, for example, evidence-based national guidelines support healthcare professionals in making informed clinical decisions [4], political priorities are often less strongly grounded in empirical evidence [5].

It has been demonstrated that many priority-setting procedures extend beyond purely technical and administrative considerations. For example, a climate of trust—characterized by stakeholders’ access to relevant information and evidence—is crucial for fostering strong relationships among stakeholders and is essential for achieving consensus in complex priority-setting decisions [3, 6–9]. Legitimacy is therefore central to priority setting, encompassing both *procedural legitimacy*—how and by whom decisions are made—and *substantive legitimacy*—the extent to which the outcomes and content of decisions are accepted [10].

Although priority setting is essential for balancing resources to promote equity and improve population health, it can be an emotionally demanding decision-making process [11, 12], particularly for clinicians responsible for implementing prioritization decisions. In practice, department managers are often expected to communicate multiple and sometimes competing priorities within their clinical units and to take the lead in implementing decisions that may be unpopular, especially those associated with budgetary constraints or service reductions [13]. Regardless of whether department managers have been directly involved in priority-setting decisions that affect their patients, they remain accountable for ensuring continuity of care, coordination, and patient safety [14].

### Priority-setting frameworks

In priority setting, frameworks can contribute by enhancing procedural transparency and serving as an initial conceptual step—whether as a model, tool, or guiding structure—to support decision-making. Such frameworks may comprise a set of dimensions addressing both process and outcome elements, thereby guiding the planning and implementation of priority-setting activities [6, 15, 16]. They may also take the form of feature-based approaches informed by healthcare managers, such as the 19-point checklist developed by Smith et al., which identifies key elements associated with high performance in priority setting [17]. According to Seixas et al. [16], frameworks are of three major types: (1) Program Budgeting and Marginal Analysis (PBMA); (2) Health Technology Assessment (HTA); and (3) Multiple-criteria value assessment.

Although many priority-setting frameworks are ambitious in their aims—such as improving the allocation of limited resources and maximizing population health—substantial challenges remain in identifying appropriate health outcome measures to assess their impact [18, 19]. A review by Kapiriri and Razavi examining multiple priority-setting models and tools found that their routine use in practice remains limited and highlighted ongoing concerns regarding how to increase their adoption. The review further emphasized the need to systematically develop, evaluate, and synthesize the strengths and limitations of different approaches to enable policymakers to select models that are best suited to their specific priority-setting contexts [20]. While priority-setting frameworks are intended to facilitate difficult and often contentious decisions about priorities [2], research suggests that managers may experience distress when such decisions conflict with their professional values or managerial responsibilities, including the obligation to ensure patient safety and continuity of care within their clinical units [21]. Moreover, formal training in priority-setting frameworks does not necessarily mitigate this distress. As demonstrated by Mitton et al., such training may instead heighten awareness of ethical dilemmas, which in some cases has led managers to withdraw from their roles or assignments [21].

Sibbald et al. [6] identified ten overarching elements that can guide the planning process and support organizations in managing the complex task of priority-setting decision-making. Five of these elements relate to process considerations: stakeholder engagement, an explicit decision-making process, information management, attention to context and values, and the presence of review or appeal mechanisms. In addition, five outcome elements are expected: stakeholder understanding, shifts in priorities and resource reallocation, improved decision-making quality, stakeholder acceptance and satisfaction, and positive externalities [6]. In this article, we apply Sibbald et al.’s framework and its key elements as an analytical structure and conceptual foundation for examining the implementation of a Swedish priority-setting model, the National Model for Transparent Prioritization (NMTP) [22].

In the context of global healthcare systems facing increasing economic pressures, the introduction of procedures and tools to support priority-setting decision-making is crucial. However, it is equally important that such procedures and tools are evaluated and perceived as valuable, useful, and reliable [11, 18, 20]. The NMTP, mentioned above, was introduced as a decision-support tool in decommissioning processes within one Swedish region responsible for the financing and provision of healthcare, with the aim of facilitating decision-making, ensuring consistency, and achieving fair priorities. The aim of this study was to explore department managers’ perceptions of using the NMTP at their clinics during the implementation of a decommissioning programme. In addition, the study explores their perception of whether the model contributes to fair priority setting and to procedural and substantive legitimacy.

### National model for transparent prioritisation in Swedish healthcare

In Sweden, a relatively widely used framework for priority setting is the National Model for Transparent Prioritisation (NMTP). The first version was published in 2007. It was developed by the National Centre for Priority Setting in Health Care, which is a national knowledge centre working with research, education and policy development regarding priorities in healthcare, also supporting regions, municipalities and clinics. One of the main financers are The Ministry of Health and Social Affairs [23].

The NMTP is classified as a multi-criteria value assessment framework [16]. The model is grounded in the ethical platform established by the Swedish Parliament in 1995, which comprises three guiding principles: the human dignity principle, the needs–solidarity principle, and the cost-effectiveness principle [24, 25]. The NMTP aims to promote the systematic application of this ethical platform to ensure that healthcare resources are allocated appropriately and efficiently, with particular emphasis on those with the greatest needs. The NMTP is applied in both vertical and horizontal priority setting. Vertical prioritisation refers to prioritisation decisions within a specific disease area, such as choices between different treatment options. Horizontal prioritisation involves reallocating resources across clinics or services within a region. Horizontal priorities may also be set within individual clinics, where different professional groups jointly define priorities and develop shared rankings of services for specific patient groups—for example, in outpatient clinics or primary care centres. The NMTP is based on a qualitative assessment of severity of condition, expected patient benefit, cost-effectiveness, and strength of evidence [22]. These principles are also embedded in the national guidelines for healthcare [25]. Previous research on the NMTP indicates benefits but emphasises the need for strong managerial commitment [26, 27].

### The decommissioning case

In Sweden, the healthcare system is highly decentralised. Each of the country’s 21 politically governed regions has independent budgets and is responsible for financing and delivering the majority of healthcare services; approximately 75% of healthcare funding is derived from regional income taxes. Consequently, budget deficits and resource constraints must be managed independently by each region. This article focuses on Region Dalarna, which has approximately 280,000 inhabitants and fifty clinics, which includes departments at five hospitals (specialized care) and 30 primary healthcare centres. The region underwent a decommissioning programme that has been described elsewhere [28, 29]. The aim of the programme was to achieve budget balance within three years through a structured decommissioning process decided by the region’s executive leadership and involving both elected politicians and public officials [30]. The NMTP was introduced as a decision-support tool during the implementation of the decommissioning programme to facilitate transparent priority setting and to achieve fair priorities [22].

### The implementation of the National model for transparent prioritisation in Region Dalarna

**Fig. 1:**
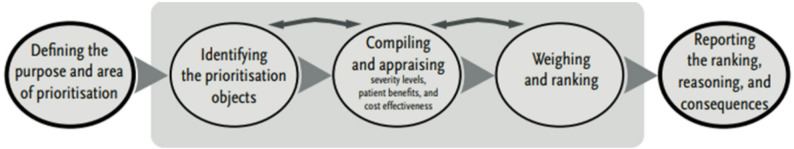
Illustration of the national model for transparent prioritisation in swedish health care

**Fig. 2:**
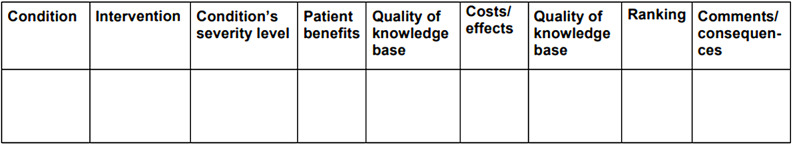
Worksheet for documenting the steps in the national model for transparent prioritization in Swedish healthcare. Source: Figures 1 and 2, The National Model for Transparent Prioritization in Swedish Health Care. National Centre for Priority Setting in Health Care 2011:4

When the decision was made to apply the NMTP more systematically in Region Dalarna, department managers and clinical units were offered education, training sessions, and ongoing support in the use of the model (98% participated). The education focused on key components of the NMTP, such as defining the purpose of prioritisation and identifying prioritisation objects, Fig. 1. A structured worksheet covering the different steps of the model was used both during training sessions and in the actual prioritisation processes to support a systematic approach to the prioritisation process. Discussions addressed the compilation and qualitative weighting of severity of condition, expected patient benefit, and costs in order to arrive at a final ranking of services or interventions, Fig. 2. In addition to formal training, continuous support was provided to department managers and their clinical units over the implementation period, for example through participation in clinic leadership meetings. During this period, a dedicated support team—consisting of a medical adviser and an NMTP expert—visited and assisted multiple clinics in their priority-setting work using the NMTP. These support sessions typically lasted 90–120 min.

## Methods

A convergent mixed methods design based on a survey and interviews.

### Design

A mixed-methods approach was adopted to obtain a comprehensive and in-depth understanding of department managers’ perceptions of the use of the NMTP and its legitimacy [31]. In a previous study [32], a survey was used to explore healthcare managers’ overarching perceptions during the implementation of the decommissioning programme. A subset of survey items focused specifically on the use and legitimacy of the NMTP, as detailed in Additional file 1. The data relating specifically to the NMTP constitute the empirical basis of the present study and have not previously been analysed or published. It was collected concurrently with the data for the published article examining the broader context of the decommissioning programme, however as a standalone element [32]. Combining quantitative and qualitative data enables the exploration of more complex research questions and provides a broader understanding of both *what* was perceived and *how* these perceptions were formed [33, 34].

### Survey development

A section of six items in the questionnaire (formulated as statements) focused on how department managers used the NMTP during the implementation of the decommissioning programme. The six items were informed by the prioritization literature [35, 36] and further refined through the interviews conducted during the development of the survey [29].

The six items were graded on a five-point Likert response scale (strongly disagree, disagree, neither, agree, strongly agree) along with the response ‘don’t know’. Twelve former department managers with knowledge of the NMTP assessed the relevance of the statements in the first draft of the questionnaire by participating in a content validity survey [37]. The participants received instructions on how to assess the relevance of the items to capture and better understand the managers’ perception of the NMTP. The participants were also asked to verbalise their thoughts about the content and language. The verbal feedback affected two items in the questionnaire which were rephrased. The I-CVI (Item-Content Validity Index) range of the items was 0.91-1.0. Table 1 reports the S-CVI (Scale-Content Validity Index), Cronbach’s alpha, and question topics for the subscale which both demonstrates very good validity.

**Table 1:** Subscales including S-CVI, Cronbach’s alpha and question topics

Subscale	S-CVI	Cronbach’s alpha	Item topics
Perception regarding the NMTP	0.98	0.94	1) Use and contribution of the NMTP a) Adoption b) Contribution to the exclusion of previously provided care c) Contribution to the inclusion of care previously not provided 2) Legitimacy of the NMTP a) Acceptance as a tool b) Justification of time spent in relation to decisions being well-informed and of high quality c) Contribution to prioritising patients with the greatest needs

### Sample and data collection

#### Participants

All department managers who had received training and utilized the NMTP participated in the survey (*n* = 33) while a selection of these participated in the interviews (*n* = 22). The interview participants were selected to represent all divisions and different geographical locations of primary care centres. In their entirety, they constitute a representative sample of all department managers who received training and utilized the NMTP.

#### The survey

The survey respondents included both department mangers responsible for patient care and department managers overseeing service departments, such as radiology and laboratory services. Department managers were invited to participate in the survey during one of their biannual departmental management meetings. Information about the survey was provided in advance through both the meeting invitation and the agenda. At the meeting, participants received both verbal and written information outlining the voluntary nature of the survey and their right to decline participation without providing a reason, including the option to refrain from attending the scheduled survey session. A total of 50 out of 54 department managers consented to participate in the original study. Of these, 33 managers who had received education in and used the NMTP in their clinical units responded to the survey questions related to the NMTP. Four department managers were not present at the meeting. Participants were informed that survey data would be handled exclusively by the research team and that results would be reported in a manner that ensured individual anonymity.

#### Interviews

The department managers selected for interviews were responsible for patient care and for decisions related to treatments and medical interventions, including the associated costs of care. A semi-structured interview guide was developed to capture department managers’ experiences of applying the NMTP in practice, Additional file 2. Of the 26 department managers invited, 22 had received training in and used the NMTP in their clinical units and were subsequently interviewed regarding their perceptions of the model. Department managers were invited to participate via email and were informed about the study’s purpose and the voluntary nature of participation. Prior to the interviews, participants were again provided with both verbal and written information about the study and their right to withdraw at any time without consequence. Written informed consent was obtained from all participants.

### Ethical approval

The interview study was approved by the regional ethics committee in Uppsala (No. 2016/504), and the survey study was also approved by the same committee (No. 2016/504B). The study was conducted in accordance with the Declaration of Helsinki.

### Analytical procedure

The analysis in a convergent mixed-methods study involves several sequential and integrative steps [34, 38]. In this study, these steps were largely informed by the approach described by Younas et al. [31]. The analytical process and resulting inferences are presented using joint displays, which have been shown to be an effective method for integrating and presenting quantitative and qualitative findings [39].

The initial steps comprised a descriptive analysis of the quantitative data derived from the questionnaire and thereafter a deductive content analysis of the qualitative interview data, using the survey questions as an analytical framework. Supporting quotations are provided to substantiate the qualitative findings. To strengthen theoretical interpretation, the framework developed by Sibbald et al. [6]—which outlines ten key elements essential for successful priority setting—was used as a knowledge base to inform and contextualize the data-driven inferences from both the quantitative and qualitative analyses. Relevant elements from this framework were incorporated into a joint display (Table 2). In the final analytical step, data-driven inferences from the quantitative and qualitative strands were compared and assessed for confirmation and complementarity [40]. Through this process of integration and by examining the alignment between empirical findings and the knowledge base, a final meta-inference was developed for each question. A comprehensive table (Table 2) summarizing all meta-inferences derived from the integrated quantitative and qualitative data, together with the relevant elements from Sibbald et al.’s framework, is presented. Our approach was not to give one data source more weight than the other, that is to prioritize one data set over another in terms of value [41]. Instead, we aimed for a more holistic picture. We analysed whether the quantitative and qualitative data were in agreement or differed and whether the qualitative data contributed with further insights into or explanations of the quantitative data.

### Findings

The presentation of results starts with a quantitative summary of the six statements. Thereafter, quantitative and qualitative findings, including the supporting quotes, are presented together under the two overarching item topics: (1) The utilisation and contribution of the NMTP (1a-1c) and (2) The NMTP´s legitimacy (2a-2c), see Table 1. Supporting quotations are numbered 1 to 22 to represent the informants meta inferences and the knowledgebase—i.e. relevant element(s) for successful prioritisation procedures, informed by Sibbald et al. [6], are presented last in the results section. A full table with all data sources combined can be found in Table 2.

### Quantitative summary

**Fig. 3:**
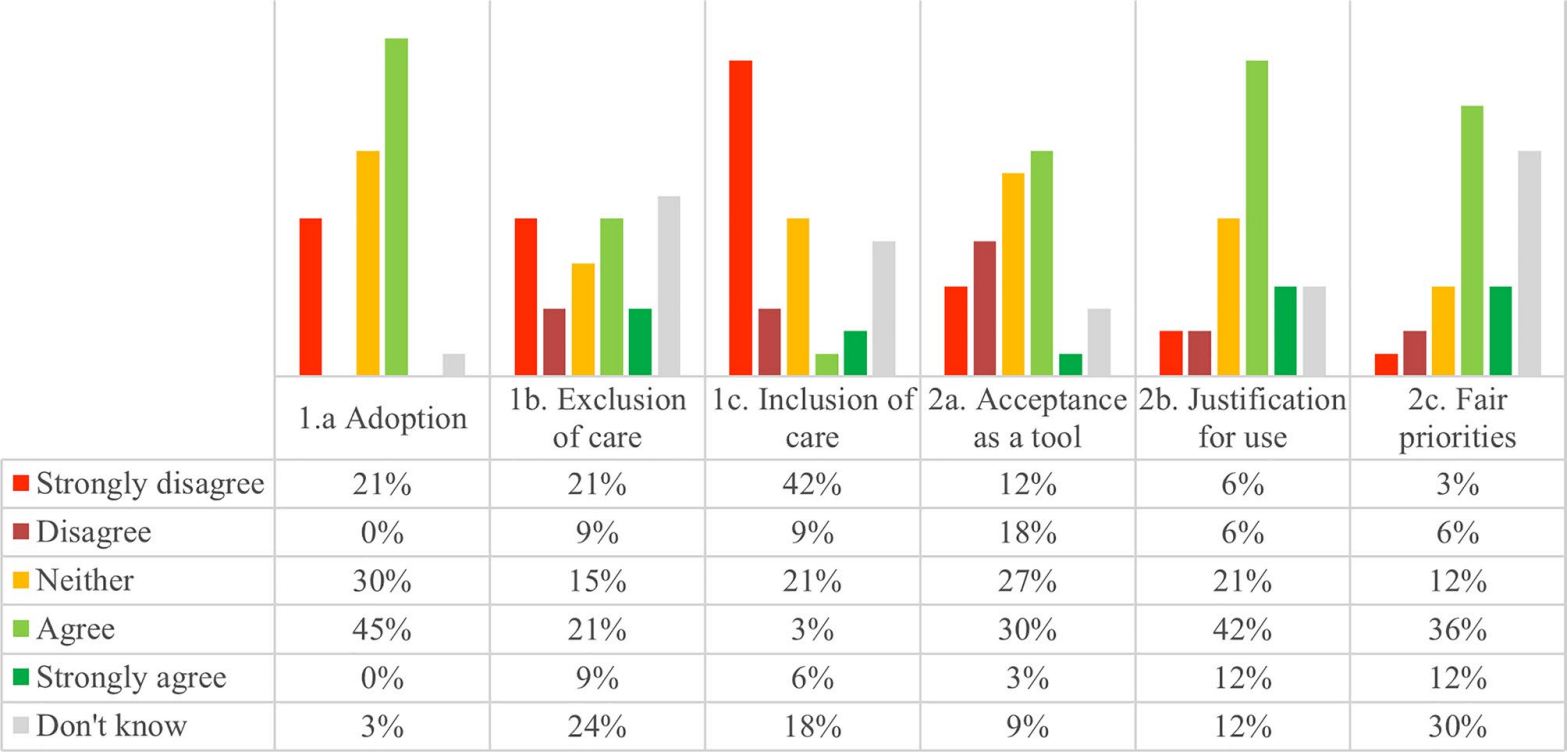
Department managers’ responses to statements about use and contribution of the NMTP (1a-1c) and legitimacy of the NMTP (2a-2c). The bars show the proportion that selected the response option

Figure 3 illustrates variation in department managers’ responses both between and within the six statements. Overall, 45% of respondents agreed or strongly agreed that the NMTP was used in their department. The highest levels of agreement were observed for statements related to legitimacy—specifically justification and contribution—with 54% and 48% of respondents, respectively, agreeing or strongly agreeing. In contrast, lower levels of agreement were reported for statements related to use and contribution. For example, only 9% of respondents agreed or strongly agreed that the use of the NMTP had led to the inclusion of care that was previously not provided. It should be noted that some questions generated a large proportion of “don’t know”-responses, indicating that the department managers found them difficult to answer or did not have enough information or experience.

### The NMTPs use and contribution: joint presentation

#### 1a. Adoption

The quantitative data shows that there was a variation in whether the department managers perceived that the NMTP was used to allocate resources in their department: almost half agreed, about one fifth strongly disagreed, while about a third were undecided (see Fig. 3). This was supported by the qualitative data that illustrated that the adoption of the NMTP varied between departments, and the extent to which the model had been used. The NMTP comprises a number of stages that necessitate a substantial investment of time, proficiency in a range of techniques and methodological expertise, which may explain why only half of the reported using it. Three supporting quotations exemplify the variation in use and point to some obstacles:

1. *“We have worked with it, identified different types of patients and set out what we need to do with them”. (Informant 16)*
2. *“It’s just not the right time right now, we’ve had so many other projects”. (Informant 14)*
3. *“We’ve just skimmed the surface, but it’s really challenging. I think you have to put in a lot more work to get into the model and past this initial hurdle, which is pretty difficult. You can easily get stuck at the beginning.” (Informant 10)*

#### 1b. Contribution to exclusion of care

The quantitative data shows that a third of the department managers had used the NMTP to exclude care that was previously provided, while an equal proportion of managers had not used the NMTP to that purpose. A quarter of department managers responded “don’t know”, indicating that it was difficult for them to answer (see Fig. 3). This picture was supported by the qualitative data illustrating that the NMTP proved to be a beneficial tool in certain departments, facilitating the identification of prioritisation objects or processes assessed as being withdrawable. Three supporting quotations exemplify the variation in perceptions among department managers and illustrate their reasoning:

1. *“We also identified a few things we previously provided, but we probably have to stop doing some of them […] we’ve stopped offering check-ups to some patients because we don’t think they need them” (Informant 9).*
2. *“We shouldn’t start diagnosing and treating certain patients. No. They have too high-level of function. We came to that conclusion.” (Informant 20).*
3. *“There are lots of workshops that are pointless. You can decide what to do intuitively without the NMTP.” (Informant 11).*

#### 1c. Contribution to inclusion of care

The quantitative data shows that a significant proportion of department managers perceived that the NMTP had not contributed to introduction of new care services, while only 9% agreed (see Fig. 3). This is reasonable since the model was introduced during a decommissioning programme, although it also aimed at more long-term efficiency improvements, potentially conceivable by introducing new methods. In line with this, the qualitative data focused more on the potential use of the NMTP for inclusion of care, giving examples of when it had resulted in replacement of outdated procedures and saying it could be useful:

1. *“A new approach has been introduced that allows patients to have an X-ray directly, so they can continue their treatment straight away.” (Informant 7).*
2. *“It could be useful for including new treatments in our department.” (Informant 16).*

### The NMTP’s legitimacy: joint presentation

#### 2a. Acceptance as a tool

The quantitative data illustrates that approximately one-third of the department managers indicated that the NMTP was accepted, while one-third stated it was not. Almost one-third remain undecided regarding the acceptance of the NMTP in their respective departments (Fig. 3). The qualitative data provided additional explanation in that the NMTP was a well-known method for discussing and establishing priorities in some departments while being perceived as complex by other departments, where it was difficult to justify its use and define its purpose. The local priority-setting decisions were not always aligned with e.g., national rules, strategies or guidelines and there was a lack of horizontal prioritization between departments. Four supporting quotations illustrate the variation:

1. *“My group was already familiar with this way of thinking, so we quickly got on board and have continued to do so”. (Informant 21)*
2. *“We sat there together and scored those points […]. We discussed, argued, and still came to an agreement, so it was probably effective.” (Informant 11).*
3. *“I can’t see clearly that it’s being used horizontally.” (Informant 16).*
4. *“We’ve had enough. Why should we use yet another model?”. (Informant 17)*

#### 2b. Justification for use

The quantitative data indicated that the majority of managers reported that the time spent was justified. However, one in five managers expressed uncertainty, and one in ten reported that the time spent was not justified (Fig. 3). The justification was further explained in the qualitative data. The NMTP contributed to decisions grounded in evidence although it required a substantial investment of time and expertise, as well as a comprehensive understanding and knowledge. Concerns were raised regarding the lack of necessary knowledge to ensure that the work was carried out to an adequate standard. Four supporting quotations illustrate the variation:

1. *“And we thought it was a good use of time to actually sit down one day and discuss this.” (Informant 9).*
2. *“It takes a lot of time, so it makes you wonder whether the work is actually worth the time. In a way, this is a question of how you prioritise your time.” (Informant 19).*
3. *“It’s really important to have the right skills and knowledge to do this job well. If we’re going to use this NMTP, we really need to get our heads around it. The thing is the model just doesn’t work well because people don’t have the right skills or experience.” (Informant 21).*
4. *(…) there are pros and cons to this. We need to think about the different aspects of it, like what we are doing, how we are doing it, why we are doing it and whether we have to do it.” (Informant 16).*

#### 2c. Fair priorities

The quantitative data shows that the majority of department managers reported that the NMTP contributed to the prioritisation of patients with the greatest need. However, a significant proportion of don’t know answers were reported, indicating that about a third of department managers found it difficult to assess. Similarly, the qualitative data indicated that the department managers perceived the NMTPs’ contribution in prioritising patients with the greatest need to be valuable. This enabled the departments to review their practice and to reallocate resources among different patient groups. Two supporting quotations illustrate the department managers’ perception:

1. *“Those with high-priority needs are seen more promptly, but those with low-priority needs are not seen at all.” (Informant 11).*
2. *“It helps us to offer cost-effective treatment to patients who previously had no access to any treatment at all, to patients who really need it.” (Informant 1).*

### Meta inferences and knowledgebase

The meta inferences (MI) built from quantitative and qualitative data and supporting quotes are collected in a joint display (Table 2) with all results. In this table, the knowledge base drawn from Sibbald et al. is also included. The meta inferences will be elaborated on in the discussion section, but in short, they point to the use of the NMTP being overshadowed by other work (MI 1a) with minor impact on both exclusion and inclusion of care (MI 1b-1c), for example due to time issues and high initial thresholds. Acceptance was determined to some extent by previous experience (MI 2a), but also by clashes with national regulation (MI 2a) and justification was reduced due to adequate knowledge and lack of relevant research (MI 2b), leading to different views on its contribution to fair priorities (MI 2c).

## Discussion

This paper presents findings from a mixed-methods study exploring department managers’ perceptions of the use and legitimacy of a priority-setting model—the National Model for Transparent Prioritization (NMTP)—within their respective departments. The discussion integrates the study’s meta inferences (MIs) with relevant elements from the knowledge base developed by Sibbald et al. [6]. The implementation of the NMTP encompassed several elements identified by Sibbald et al. as pivotal for achieving effective priority setting.

The findings indicate that the implementation and use of the NMTP were insufficient in more than half of the departments, suggesting that the primary objective of introducing the model—namely, to enhance consistency in the decommissioning decision-making process—was not fully achieved. The reasons for non-implementation and underutilisation varied and included constraints related to limited time and a high initial threshold for use (MI 1a). Furthermore, the NMTP appeared to have only a limited impact on both the exclusion of previously provided care and the inclusion of care that had not previously been offered by the clinics, as reflected in MIs 1b and 1c. With regard to legitimacy, acceptance of the NMTP varied across departments, with the majority of department managers expressing some degree of reluctance toward its adoption (MIs 2a–2c). The failure to introduce and fully implement the NMTP may be attributable to several factors. One potential explanation is a lack of genuine *stakeholder engagement and commitment* among department managers, as suggested by Sibbald et al. [6]. In addition, departments may have been overwhelmed by the demands associated with implementing the decommissioning programme, leaving limited time and capacity to focus on the NMTP. Throughout the programme, clinics were required to address numerous decisions, organisational changes, and practical challenges. Consequently, many department managers and staff perceived the use of the NMTP as overly time-consuming. Thus, limited stakeholder engagement, high thresholds for use, and time constraints suggest that the procedural conditions necessary for legitimate priority setting were only partially fulfilled during the decommissioning programme.

The legitimacy of the NMTP as a prioritisation-support tool was perceived differently across departments. When interpreted through the knowledge base developed by Sibbald et al. [6], the NMTP appeared to *improve the quality of decision-making* by enhancing transparency, structure, and documentation through the provision of an *explicit process*. The model facilitated consideration of relevant values and contextual factors related to previously provided treatments and investigations, which in some departments led to *shifts in priorities and the reallocation of resources*. Despite these positive effects on priority-setting decisions, the implementation and use of the NMTP required substantial investments of time and expertise, as well as access to a broad range of specific knowledge, including evidence related to the prioritisation object and its alternatives. Divergent perceptions among department managers may partly reflect differences in prior experience and knowledge, or the absence thereof. These reported obstacles may also have influenced managers’ willingness to engage with, implement, and use the NMTP—an issue described by Sibbald et al. [6] in terms of *stakeholder acceptance and satisfaction*. The need for robust evidence has been noted before [26, 27]. Notably, acceptance of the NMTP remained relatively low, despite the fact that most department managers perceived the model as contributing to more appropriate and equitable priorities and considered the time and resources required for its use to be justified. This apparent discrepancy—between relatively low acceptance of the model and positive perceptions of its contribution to fair and equitable priorities—suggests a divergence between procedural and substantive legitimacy. While the outcomes of priority-setting were often viewed as justified, the processes through which these outcomes were reached were not consistently experienced as legitimate.

A challenge emphasised by department managers was the difficulty of aligning local priority-setting decisions with national policies while ensuring that local decision-making adequately reflected the region’s constrained economic circumstances. Such tensions may undermine both procedural legitimacy, by constraining local discretion and deliberation, and substantive legitimacy, by complicating the justification and acceptance of prioritisation outcomes at the clinical level. This challenge may partly stem from the substantial number of reports, guidelines, and policy directives issued by national authorities to healthcare departments. Previous research indicates that such national reports, decisions, and policies can at times create conflicts and tensions with departments’ own priority-setting processes [42]. Furthermore, scholars have noted that unstable, unstructured, and ad hoc forms of governance at the national level may impede or undermine local priority-setting decisions. Addressing this challenge requires national authorities to provide a coherent and persuasive narrative that can justify unpopular local decisions and thereby strengthen their legitimacy [19].

Department managers indicated that the NMTP was not utilised for horizontal priority setting—for example, across units, departments, or at the regional level—to enable the review and reallocation of historically allocated resources. Moreover, the Swedish healthcare system is characterised by a high degree of decentralisation, with 21 politically governed regions operating independent budgets and establishing their own priorities. However, the increasingly constrained budgets faced by nearly all regions have raised questions about the effectiveness of this system. A key challenge is how to ensure equitable healthcare for the Swedish population within the context of these fiscal constraints.

Smith et al. identify a paucity of research on the implementation of priority-setting frameworks, particularly within the context of organisational change processes, and on how such procedures are situated within broader organisational and policy environments. They further highlight the need to assess the usability of these frameworks, identify barriers to their implementation, and clarify their role within wider systemic contexts [43]. The use of the NMTP during the implementation of the large-scale decommissioning programme examined in this study illustrates how a priority-setting framework can be embedded within, and shaped by, a broader organisational context. Despite the heterogeneity of contexts and strategies within healthcare systems, this study has identified universal barriers and opportunities related to the implementation of priority-setting tools, such as the NMTP. The findings demonstrate that there is a necessity for specific factors, including adequate knowledge, sufficient time, and coordination across organizational levels in priority-setting. At the very least, conditions should be relatively similar in local tax-financed health care systems.

Finally, numerous challenges have been reported regarding the use of priority-setting frameworks in prioritisation initiatives. Scholars have also documented their limited application in real-world settings. One key issue identified is the lack of comprehensive guidance on the selection, implementation, and evaluation of such frameworks [11, 18–20]. Another recurring concern is that, despite substantial investments of time and resources in introducing and implementing formal priority-setting processes, these efforts often lack sustainability. Such processes have been shown to be used most intensively during periods of financial deficit [8], as was the case in some departments in Region Dalarna during the implementation of the extensive decommissioning programme. Researchers have also highlighted ongoing uncertainty regarding the practical impact of priority-setting models. While these tools may support more transparent and deliberative decision-making processes, their influence on the actual allocation of services remains unclear [19].

Some limitations of the study should be acknowledged. The number of department managers who participated in both the interviews and the survey is unknown; consequently, it is possible that the same individuals contributed to both the qualitative and quantitative data, as the survey was conducted anonymously. In addition, there is a possibility that department managers provided socially desirable responses during the interviews, although no specific indications of this were identified.

## Conclusion

The implementation of priority-setting models in decommissioning processes should be carefully planned and clearly integrated into broader resource allocation initiatives to ensure adherence and effective use. Given that priority-setting models can be time-consuming, their successful application requires substantial investments of time and access to relevant knowledge. The overarching objective of the NMTP is to embed the use of ethical guidelines in decision-making and to ensure that resources are allocated to those with the greatest needs. This represents an urgent and long-standing challenge, and for the NMTP to fulfil this role, the model must be effectively embedded both within regional healthcare systems and in the interaction between national and regional levels of governance.

**Table 2:** Joint display with all results

Statement	Quantitative inference	Qualitative inference	Meta inference	Knowledge base
The National Model for Transparent Priorities is used to allocate resources in the department I lead.	45% agrees or strongly agrees.	Variation in use, requires substantial investment.	The decision to systematically use the NMTP to facilitate and bring consistency in the decommissioning-making process did not reach its full intention. Just under half of department managers report that NMTP was used to allocate resources in their departments. Despite the overarching decision to use the NMTP systematically in the departments, it was perceived as having been overshadowed by other work.	Engage stakeholders effectively and strive for genuine commitment and adequate engagement (Stakeholders Engagement).
The utilisation of the National Model for Transparent Prioritization has resulted in the exclusion of care that was previously included within the range of services provided.	30% agrees or strongly agrees.	Beneficial tool in certain departments.	Department managers reported various perceptions and only a third of the department managers reported withdrawal of services that had previously been a part of their service. NMTP provided a framework for decision-making and documentation of the process. Discussions were described as fruitful, and the NMTP was instrumental in facilitating these discussions.	A review of previous decisions regarding allocation of resources may be viewed as a success (Shifted priorities and/or Reallocated Resources).
The utilisation of the National Model for Transparent Prioritization has resulted in the inclusion of care that was previously not included within the range of services provided.	9% agrees or strongly agrees.	New services have been introduced.	NMTP had a minor impact on the decision-making that led to the introduction of new treatments and services. The primary objective of the decommissioning programme was to address the region’s economic challenges, which could have influenced the feasibility of implementing new services.	Institutional learning could increase compliance with decisions and develop more consistent decision-making processes (Improved Decision Making Quality).
It is my experience that the National Model for Transparent Prioritization has been accepted as a tool for prioritisation and resource allocation in the department I lead.	33% agrees or strongly agrees.	Well-known but perceived as complex.	The NMTP´s acceptance as a tool was influenced by prior experience and knowledge. A notable number of department managers acknowledged the absence of both context and a plan for sustainable use of the NMTP. The acceptance of NMTP varied between departments, with a tendency towards lower levels of acceptance. The issue of local priority-setting decisions being overridden by government regulations and guidelines was recognized as problematic.	Priority-setting procedures ought to be met with a willingness to participate (i.e., buy-in) and a certain degree of contentment to be successful (*Stakeholders Acceptance and Satisfaction)*. A predetermined process brings transparency, trust and confidence in the process *(Use of Explicit Process*). It is imperative to incorporate a revision process to ensure optimal outcomes and to prevent any potential shortcomings or disagreements. Furthermore, the refinement of rules and requirements in decision-making processes is crucial (Revision or Appeal Mechanism).
The time and resources required for priority-setting, supported by the National Model for Transparent Prioritization, are justified by the fact that the priorities will be more well-founded and of a high quality.	54% agrees or strongly agrees.	Contributes to grounded decisions but concerns about lack of knowledge and evidence.	A considerable proportion of the department managers expressed that the NMTP contributed to an improvement in the quality of the department’s priorities. However, the absence of adequate knowledge to facilitate optimal decision-making was identified as a potential risk, along with the scarcity of research on the priority subjects.	Decision-making quality is ensured through the appropriate use of available evidence and compliance to the described process (Improved Decision Making Quality).
The decisions made about priorities, with the support of the National Model for Transparent Prioritization, result in patients with the greatest need being given the highest priority in the department I lead.	48% agrees or strongly agrees.	Valuable to prioritize the right patients.	The NMTP was perceived as beneficial by almost half of the department managers in identifying patients with the greatest need, and valuable in the context of priority-setting processes, facilitating a fair reallocation of resources. Several department managers encountered difficulties in evaluating this measure. Furthermore, a minority expressed skepticism regarding NMTP’s contribution to this outcome.	Priority setting decisions should be based on clear value choices and the reasons for these choices should be made explicit (Consideration of Values and Context). A reassessment of previous decisions regarding the allocation of resources may be perceived as a success (Shifted priorities and/or Reallocated Resources).

## Supplementary Information

Below is the link to the electronic supplementary material.

Supplementary Material 1

Supplementary Material 2

## Data Availability

The datasets used and/or analysed during the current study are available from the corresponding author on reasonable request.
