# Supplementary material for: Department managers’ perceptions of a priority-setting model in a local healthcare organisation: a mixed-methods study
Source: BMC Health Serv Res. 2026 Mar 31;26:496. doi: 10.1186/s12913-026-14451-z (PMC13063958; doi:10.1186/s12913-026-14451-z)

Appendix 1.

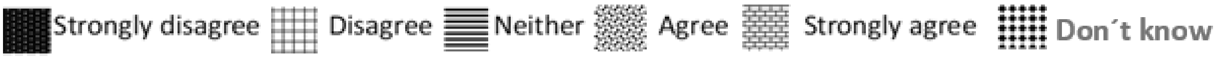

The National Model for Transparent Priorities is used to allocate resources in the department I lead

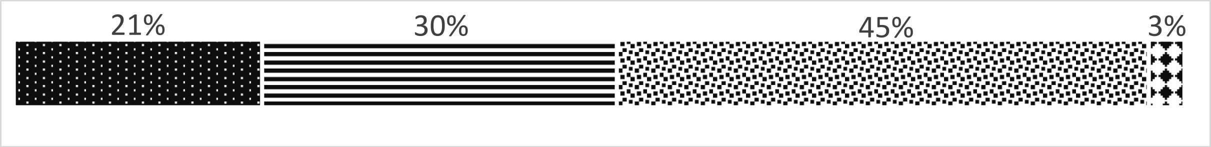

It is my experience that the National Model for Transparent Prioritization has been accepted as a tool for prioritisation and resource allocation in the department I lead

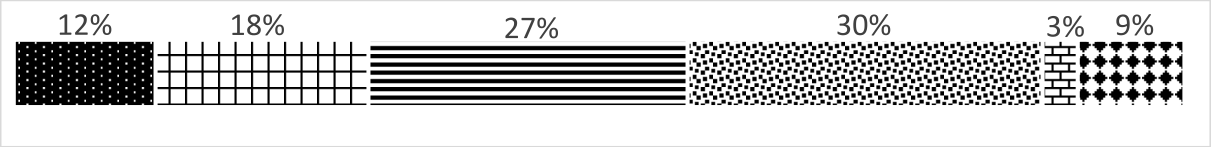

The time and resources required for priority-setting, supported by the National Model for Transparent Prioritization, are justified by the fact that the priorities will be more well-founded and of a high quality

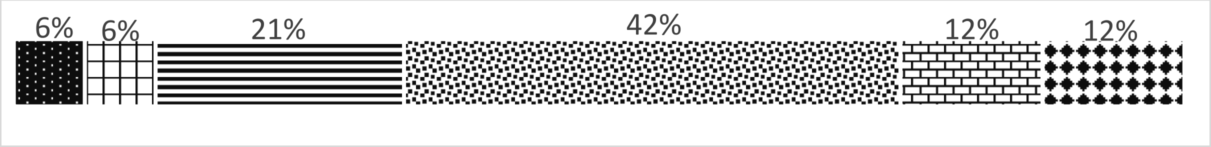

The decisions made about priorities, with the support of the National Model for Transparent Prioritization, result in patients with the greatest need being given the highest priority in the department I lead

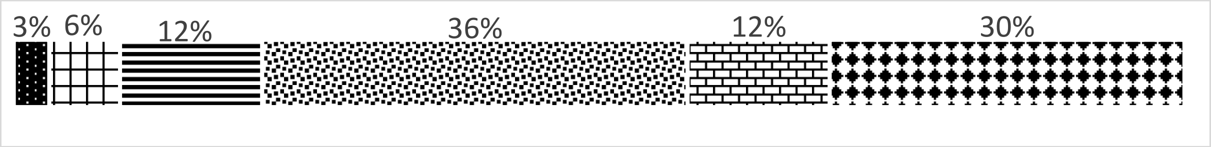

The utilisation of the National Model for Transparent Prioritization has resulted in the exclusion of care that was previously included within the range of services provided

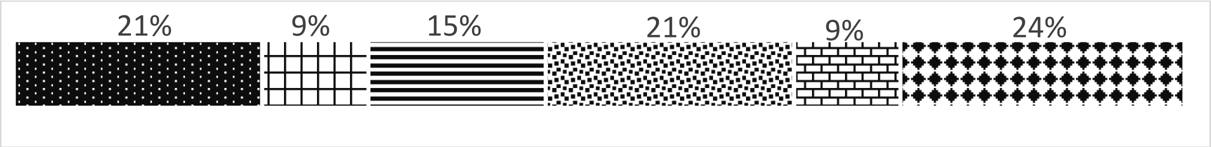

The utilisation of the National Model for Transparent Prioritization has resulted in the inclusion of care that was previously not included within the range of services provided

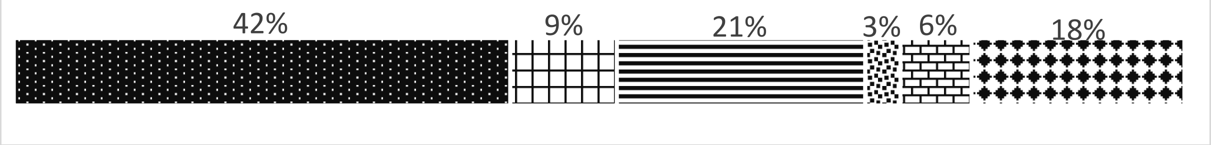

Supplement: Supplementary file 1 — Supplementary Material 1 [file 12913_2026_14451_MOESM1_ESM.pdf]
