# Supplementary material for: Department managers’ perceptions of a priority-setting model in a local healthcare organisation: a mixed-methods study
Source: BMC Health Serv Res. 2026 Mar 31;26:496. doi: 10.1186/s12913-026-14451-z (PMC13063958; doi:10.1186/s12913-026-14451-z)
Supplement: Supplementary file 2 — Supplementary Material 2 [file 12913_2026_14451_MOESM2_ESM.pdf]

Additional file 2. Interview guide

The National Model for Transparent Prioritisation (NMTP)

2b. Implementation of the NMTP

How has the NMTP been received as a tool for prioritization and resource allocation at the clinic you lead? How do you perceive the region´s efforts to introduce the NMTP?

2c. Results of working with the model

What does the NMTP contribute? How has the NMTP affected the range of care in your organisation? Are more of the right interventions being done, have resources been shifted, have indication limits been moved? Has working with the NMTP resulted in you starting to offer care that was not previously included in your range of services/ceased to offer care that was previously included in your range of services?

Do you have any examples of when the NMTP has contributed to patients with the greatest need for health care being prioritized? Do you have an example of when the NMTP has contributed to the efficient use of resources by discussing and influencing decision-making on the cost-effectiveness of measures (patient benefit/effect of measures)?

2d. Quality of work

What are your experiences of the NMTP as a tool for prioritization? What strengths and weaknesses do the NMTP have at various levels in the organisation, such as for prioritising and restructuring resources within your organisation's when allocating resources externally between operations and divisions?

What is your opinion on the knowledge base and ranking lists produced during the prioritisation work in the activities you lead? Quality of e.g. severity assessment, level of evidence and health economics?

2e. Resources

How do you perceive the time and resources required to work according to the NMTP?

2f. New learnings, experiences and insights

How do you view the applicability of the NMTP in the future? Are any changes needed?

Ending

Is there anything in particular that you thought about before or during the interview?
